# Effects of Carbon Dioxide Aerosols on the Viability of *Escherichia coli* during Biofilm Dispersal

**DOI:** 10.1038/srep13766

**Published:** 2015-09-08

**Authors:** Renu Singh, Ajay K. Monnappa, Seongkyeol Hong, Robert J. Mitchell, Jaesung Jang

**Affiliations:** 1 School of Mechanical and Nuclear Engineering, Ulsan National Institute of Science and Technology (UNIST), Ulsan 689-798, S. Korea; 2 School of Life Sciences, Department of Biological Sciences, Ulsan National Institute of Science and Technology (UNIST), Ulsan 689-798, S. Korea

## Abstract

A periodic jet of carbon dioxide (CO_2_) aerosols is a very quick and effective mechanical technique to remove biofilms from various substrate surfaces. However, the impact of the aerosols on the viability of bacteria during treatment has never been evaluated. In this study, the effects of high-speed CO_2_ aerosols, a mixture of solid and gaseous CO_2_, on bacteria viability was studied. It was found that when CO_2_ aerosols were used to disperse biofilms of *Escherichia coli*, they led to a significant loss of viability, with approximately 50% of the dispersed bacteria killed in the process. By comparison, 75.6% of the biofilm-associated bacteria were viable when gently dispersed using Proteinase K and DNase I. Indirect proof that the aerosols are damaging the bacteria was found using a recombinant *E. coli* expressing the cyan fluorescent protein, as nearly half of the fluorescence was found in the supernatant after CO_2_ aerosol treatment, while the rest was associated with the bacterial pellet. In comparison, the supernatant fluorescence was only 9% when the enzymes were used to disperse the biofilm. As such, these CO_2_ aerosols not only remove biofilm-associated bacteria effectively but also significantly impact their viability by disrupting membrane integrity.

Biofilms are complex bacterial community structures attached to a surface and connected via extracellular polymeric substances (EPS), a matrix composed mainly of polysaccharides, proteins, and DNA, which encapsulates the bacteria^1^,^2^. Biofilms exist on a wide variety of surfaces, including leaf surfaces, living tissues, implanted medical devices, membranes, ship hulls, and heat exchangers^3^,^4^,^5^,^6^,^7^,^8^. They not only cause economic losses^7^, but also present a public health hazard. In fact, based upon the U.S. Centers for Disease Control and Prevention (Atlanta, GA, USA), biofilms are associated with 65% of bacteria-borne infections and diseases in humans^5^. Part of the reason for this is that the bacteria present within biofilms are much more resistant to antibiotics, disinfectants^9^ and the host immune system effectors^10^.

From a commercial perspective, biofilms are one of the leading causes of technical failure in many industrial systems, such as cooling towers, heat exchangers and pipelines in the oil industry^7^. They cause biofouling and reduce membrane life times in water and waste water treatment plants^11^ via deterioration and corrosion of pipes and metal surfaces. Therefore, the control or removal of biofilms is critical within a wide variety of fields and applications, with many studies being conduced^2^,^7^. For example, antimicrobials or biocides have been incorporated into, or coated onto, the surface of materials^2^. Biocides or disinfectants, such as chlorine, ozone, hydrogen peroxide, and peracetic acid, have also been used to inactivate biofilms and their bacteria^12^. However, disinfectants generally do not completely penetrate the biofilm matrix and, thus, may still permit physically intact biofilms even with treatment^12^.

Therefore, physical or mechanical methods are often required to remove biofilms from a surface. These methods include electric currents^13^, laser irradiation^14^, ultrasonic vibration^15^, liquid micro-jets^16^ and high-pressure water sprays^17^. Some limitations exist for these, however. Electrical fields and laser irradiation are limited to relatively small areas^7^, and micro-jets based on drag forces are not effective at removing micrometer-sized particles^18^. High-pressure water sprays can effectively remove biofilms due to their high momentum, but the underlying substrate can be damaged by this momentum if it is not sufficiently durable.

Recently, our group presented a novel CO_2_ aerosol technique to remove *Escherichia coli* biofilms^19^. This technique employed periodic jets of carbon dioxide aerosols, a mixture of solid and gaseous CO_2_, which were generated by the adiabatic expansion of high-pressure CO_2_ gas through a nozzle. We demonstrated that this CO_2_ aerosol technique was very effective at removing biofilms from several surface materials with removal efficiencies ranging from 93.2% to 99.9% when treatment times of 40–90 s were employed^20^. Furthermore, the removal efficiencies measured from independent samples showed very small variations (generally less than 5%) when using the optimized conditions.

This CO_2_ aerosol technique is similar to high-pressure water sprays in that biofilms are removed primarily by mechanical impact or momentum transfer, but the momentum that is delivered to the surface is much smaller in the CO_2_ aerosol technique and, hence, a negligible amount of damage to the surface occurs. On the other hand, the momentum of the high-speed solid CO_2_ particles within the aerosols may not be negligible for the bacteria present within the biofilm considering that the generated solid CO_2_ diameter ranges from 0.4 to 9.6 μm with the peak of 0.7 μm^21^ and, therefore, may affect their viability. This is a particularly critical characteristic that needs to be evaluated if this technique is to be widely applied as the dispersal of bacteria into the air can be a significant environmental and health concern, especially if they are pathogenic.

In this study, consequently, we investigated the effects of the CO_2_ aerosol treatment on the viability of *E. coli* XL1-Blue biofilms. This bacterium was selected since it is non-pathogenic and has been used extensively in biofilm studies by many groups^22^,^23^,^24^,^25^. Using multiple analytical tools, including confocal microscopy and scanning electron microscopy (SEM) to visualize the biofilms before and after the treatment and flow cytometry combined with colony forming units (CFU) measurements to assess the bacterial viability, we show that these carbon dioxide aerosols are not only effective at removing bacterial biofilms but they also significantly reduce their viability.

## Results and Discussion

**Figure 1:**
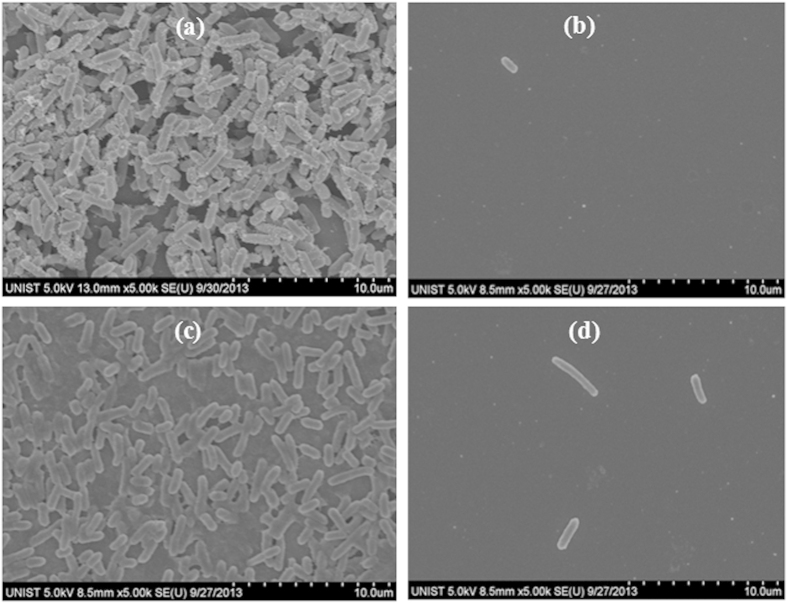
SEM images showing the effects of a treatment with either CO_2_ aerosols or hydrolytic enzymes on the *E. coli* biofilms grown for one day. (**a**) Untreated control chip showing the presence of an extensive *E. coli* biofilm on the Si surface. (**b**) Biofilm after CO_2_ aerosol treatment. (**c**) Image of *E. coli* biofilm after being soaked in 25 mM HEPES buffer for two hours. (**d**) *E. coli* biofilm after treatment with HEPES buffer containing both Proteinase K and DNase I.

SEM analysis was initially used to analyze *E. coli* biofilms grown for one day on silicon chips before and after treating them with CO_2_ aerosols or hydrolytic enzymes (Fig. 1). This figure shows that a uniform growth of *E. coli* biofilm across the silicon surface was readily apparent for the control chip (Fig. 1a) and that this biofilm was effectively removed after the CO_2_ aerosol treatment (Fig. 1b). For comparison, an SEM micrograph of the *E. coli* biofilm after soaking in HEPES buffer is also presented (Fig. 1c) along with an image showing the *E. coli* biofilm after treatment with both Proteinase K and DNase I (Fig. 1d). As these two enzymes hydrolyze the protein and DNA present within the EPS, treatment of the biofilms with both of these results in a gentle dispersion of the bacteria.

**Figure 2:**
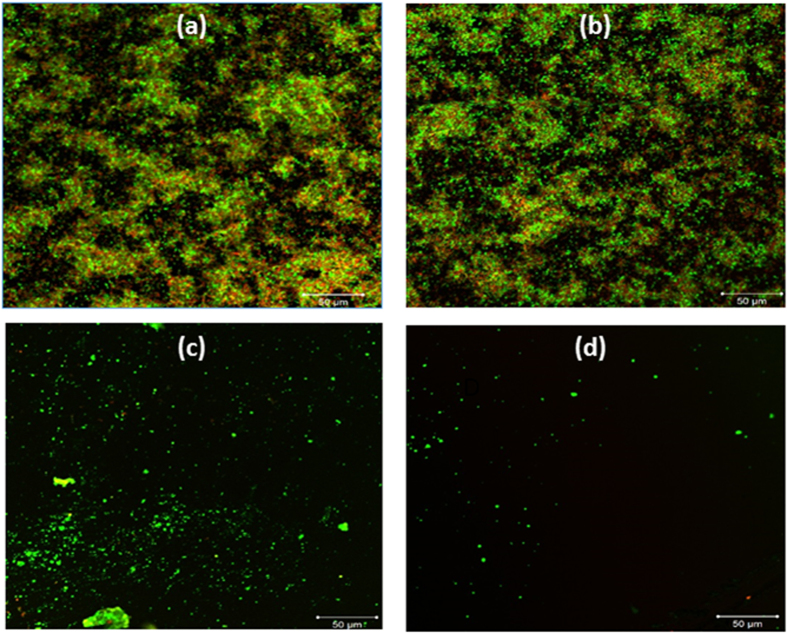
Confocal microscopic image of one-day grown *E. coli* biofilms stained with the BacLight stain (SYTO-9 and propidium iodide) after their respective treatments. (**a**) Untreated control biofilm. (**b**) HEPES soaked biofilm. (**c**) *E. coli* biofilm after treatment with Proteinase K and DNase I. (**d**) *E. coli* biofilm treated with CO_2_ aerosols. The scale bars are 50 μm.

To analyze these results deeper, particularly with regards to biofilm viability, we stained the biofilms with a BacLight stain (Invitrogen, USA) containing both SYTO9 and propidium iodide (PI), which labels the live cells green and the dead cells red, respectively. Figure 2a shows the fluorescent image of the untreated *E. coli* biofilm. Although not quantitative, the greater prevalence of green fluorescence suggests that the majority of the culture was viable, a finding that also appears true of the HEPES-treated biofilm (Fig. 2b). Treatment of the biofilm with either hydrolytic enzymes (Fig. 2c) or the aerosols (Fig. 2d), however, led to a significant decrease in both fluorescent signals, affirming the findings of Fig. 1 where a significant number of the bacteria cells were removed by both of these treatments.

**Figure 3:**
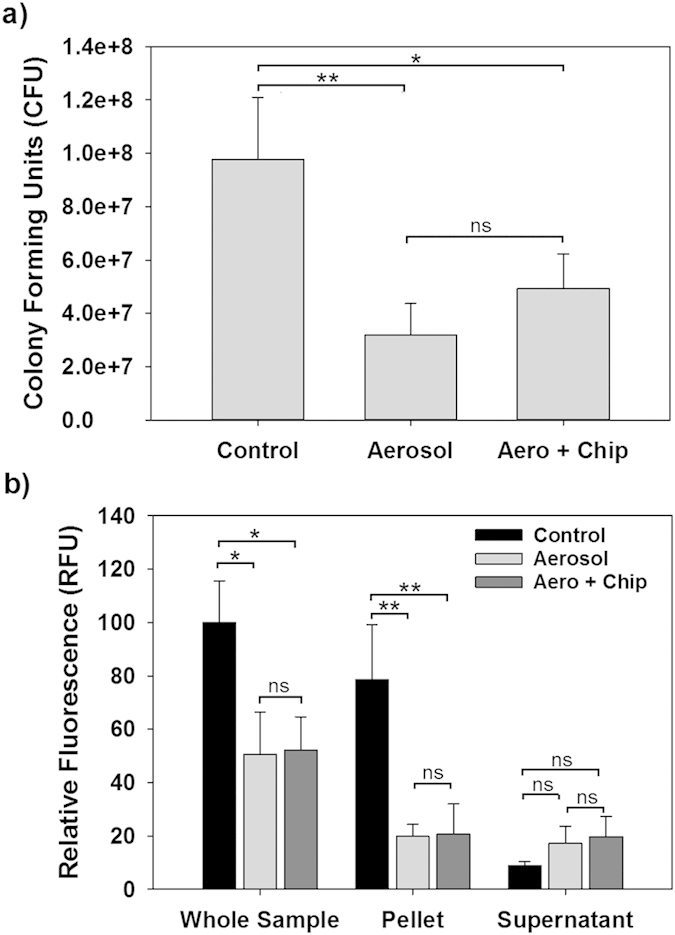
Viable number of *E. coli* present within the CO_2_ aerosol treated samples. (**a**) Colony forming units determined by plating out on agar plates. The samples are the Control, which was gently dispersed using a Proteinase K and DNase I treatment, the Aerosol sample, *i.e.* the bacteria dispersed by CO_2_ aerosols and captured within the flask, and the Aero + Chip sample, which includes both the flask captured bacteria and the dispersal of any remaining bacteria present on the chip using Proteinase K and DNase I. (**b**) The relative fluorescence seen in the whole sample or the constituent parts (*i.e.*, cell pellet or supernatant). The presence of nearly half of the fluorescence in the supernatant after CO_2_ aerosol treatment implies that a significant number of *E. coli* are being ruptured by this treatment. Statistical analysis was performed using one-way ANOVA followed by the Tukey post hoc test. Statistically significant results are identified with asterisks (*,** =P values < 0.05 or 0.01, respectively; ns – not significant).

Removal of biofilms with CO_2_ aerosols was previously demonstrated by our group^19^,^20^. Based upon the above images and a previous report^4^, it is clear that biofilms harbor a significant number of viable bacteria. However, the effects of CO_2_ aerosols on the viability of the dispersed bacteria have not been studied to date. To evaluate this, therefore, we collected three groups of samples: enzymatically dispersed bacteria from a biofilm using Proteinase K and DNase I (Control), the bacteria dispersed from the biofilm by CO_2_ aerosols (Aerosol) and those obtained after treatment of the biofilm first by CO_2_ aerosols followed by an enzymatic treatment of the biofilm still attached to the silicon chip (Aero + Chip). It should be noted that all the samples collected were treated subsequently with Proteinase K and DNase I to dissociate any bacterial aggregates that were present to ensure that the viable counts in Fig. 3a were correct.

For the control sample, which was dispersed using only the enzymes, the total number of viable bacteria per chip was on average 9.8 × 10^7^ CFU. By comparison, the number of viable bacteria in the aerosol-dispersed samples was 3.2 × 10^7^ CFU, or approximately 3-fold less. Inclusion of the bacteria still present on the chip after aerosol treatment increased the viable count to 4.9 × 10^7^ CFU, which is approximately half the original number. Based upon the number still attached to the chip, *i.e.*, 1.7 × 10^7^ CFU, treatment of the biofilms with aerosols removed approximately 80% of the biofilm-associated viable cells from the silicon chip surface. This value is not unexpected as the treatment area did not encompass the entire chip.

As the solid CO_2_ particles are comparable in size with the bacteria, it was presumed that their momentum and impact may be sufficient to cause damage to the bacteria and their membranes. To study this more in depth, we performed the same experiments with a fluorescent strain of *E. coli* that expresses the cyan fluorescent protein (CFP). It was hypothesized that if the cell membranes are damaged by the CO_2_ aerosols, the CFP protein would be released into the collection media, thereby increasing the fluorescence of the cell-free supernatant. This was found to be the case, as shown in Fig. 3b. Addition of Proteinase K to the sample was not a concern since GFP and related fluorescent proteins are known to be resistant to this protease^26^.

When considering the total fluorescence of each sample as shown in Fig. 3b, approximately half is lost during the aerosol treatments. Of that seen in the captured samples, however, approximately half was associated with cell pellet and the other half was found in the supernatant. Such a significant presence of CFP within the extracellular milieu indirectly confirms that the CO_2_ aerosols are disrupting the bacterial membrane integrity and helps explain the loss in viability seen in Fig. 3a. This activity of the CO_2_ aerosols can be attributed to the large momentum (mechanical impact) of the solid CO_2_ particles and high shear near the silicon surfaces due to the high-speed gas flows, which will be discussed below.

**Figure 4:**
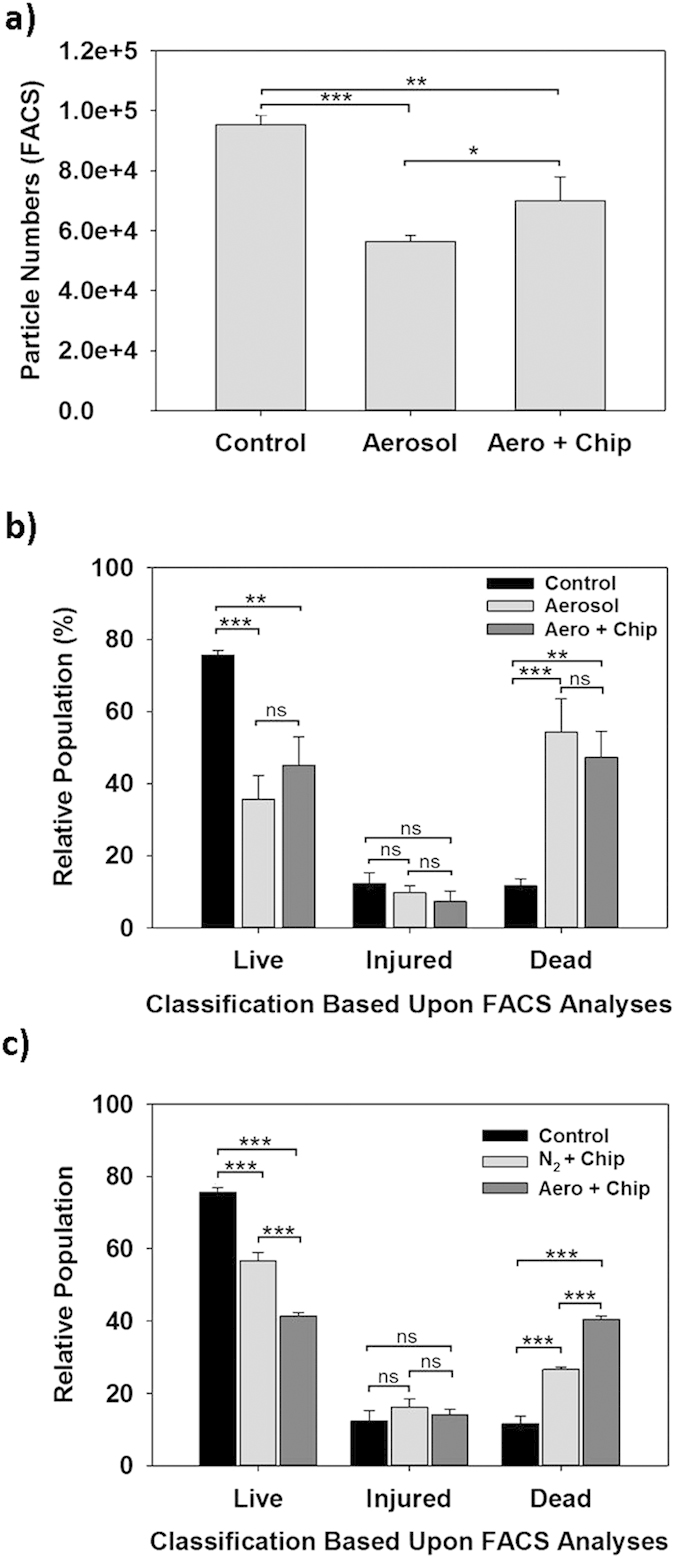
Analysis of the bacteria dispersed during the CO_2_ aerosol and enzymatic treatments by flow cytometry. The samples are the same as in Fig. 3. (**a**) Actual number of particles counted within the FSC gated region when using a fixed time of 10 seconds for each sample. (**b**) Relative populations of the cells (live, dead and injured) in each sample. (**c**) Relative populations of the cells (live, dead and injured) were dispersed by enzymatic treatment (control), N_2_ gas, and CO_2_ aerosol treatments. In these N_2_ gas and CO_2_ aerosol treatments, the dispersed bacteria were added to the bacteria present on the chip surfaces. The classification of bacterial cells into each population was performed by staining the samples with the BacLight stain (SYTO9 and propidium iodide) prior to FACS analysis. A total of 5,000 cells were analyzed for each sample, and the results show the average from three independent tests. Statistical analysis was performed using one-way ANOVA followed by the Tukey post hoc test. Statistically significant results are identified with asterisks (*, **, or *** = P values < 0.05, 0.01 or 0.0001, respectively; ns – not significant).

Although the above fluorescence results indicate that a large number of the bacteria are being injured or killed by the aerosol treatment, it still remained uncertain if the decrease in the viable counts (Fig. 3a) is due to cell death or if a portion of the bacterial population was lost due to aerosolization of the biofilm. To address this, we analyzed each of the collections (Control, Aerosol and Aero + Chip) using flow cytometry. As shown in Fig. 4a, when the same volume of sample was analyzed the particle number in the Aerosol sample was approximately 40% lower than that of the Control. However, treatment of the undispersed biofilm still present on the chip with Proteinase K and DNase I (Aero + Chip) increased this to 73%. These values show that a significant number of bacteria are not being captured, and although this contributes to the lower viability seen in Fig. 3a it does not fully account for this discrepancy.

Flow cytometry combined with Live/Dead staining is also commonly employed to evaluate bacterial viability for the samples derived from various environmental conditions, such as soil and water samples^27^. Consequently, using live and dead fractions of planktonic bacteria, we were able to gate the live and dead populations according to the FACS analyses (Fig. S1). Subsequently, using a sample number of 5,000 cells for each test condition, we were able to determine the relative live and dead populations (Fig. S2). Interestingly, there were populations that fell between these two categories and, thus, these were serendipitously branded as “injured” bacteria (Fig. 4 and S2).

Figure 4b shows that the majority of the bacteria present within the Control samples are living (76%). This is in stark contrast with the Aerosol and Aero + Chip samples, both of which harbor a significant number of dead and injured bacterial cells. These results, however, are in agreement with the results presented in Fig. 3b since 54% and 47% of the Aerosol and Aero + Chip populations, respectively, are classified as non-viable, *i.e.*, dead. By comparison, in Fig. 3b the supernatant fluorescence intensities for both samples were just below that found in the pellets. The results from both tests indicate that approximately half of the dispersed bacterial population is killed by the aerosols.

From Fig. 3a, the Aerosol sample viability was 33% while for the Aero + Chip sample it was 51%. When the relative number of particles in Fig. 4a, which represents the capture efficiency, is multiplied by the summed percent of live and injured bacteria (Fig. 4b) for each sample, we obtain a relative viability of 30% and 44% for the Aerosol and Aero + Chip samples, respectively, which is slightly lower than but similar to the colony forming units actually found (Fig. 3a). As such, the consistency between the different techniques employed in this study helps to substantiate that the CO_2_ aerosols inflict a significant amount of damage to the biofilm-associated bacterial population during treatment and that this leads to losing the viability. Moreover, it suggests that the “injured” bacteria are viable and capable of producing colonies.

The aerosols actually consist of two components – the solid CO_2_ particles and gaseous CO_2_. As mentioned above, the loss in viability is likely attributed to the large momentum associated with the high-speed solid CO_2_ particles. To study this further, and in the hope of identifying the component responsible, we also performed tests with only pressurized nitrogen gas whose stagnation pressure was the same as that of CO_2_ gas. This is based on the fact that CO_2_ and N_2_ gases would have almost the same flow patterns and shear stresses on the substrate surfaces if both gases have the same pressures. As shown in Fig. 4c, the viability of the bacteria decreased when the biofilm was treated with nitrogen alone implying viability loss due to the gas component of the aerosols. This loss was more significant when CO_2_ aerosols were used. A comparison between these two treatments shows that the viability was significantly lowered by the presence of the CO_2_ aerosols, that is, the solid CO_2_ particles and gaseous CO_2_. As such, the activity of the CO_2_ aerosols on bacterial viability can be attributed to both the large momentum (mechanical impact) of the solid CO_2_ particles and the high shear stress resulting from the CO_2_ gas flows. It should also be noted that, although a high-pressure gas treatment only damages many of the bacteria, the removal efficiency was shown previously to be very small^19^.

Although our group previously found that CO_2_ aerosols can be used to remove biofilms from various substrate surfaces, this study is the first to show that they also kill a significant portion of the bacteria during the dispersal. Using several different analytical tools and techniques, we found the aerosols significantly impact the biofilm population viability. An analysis of the supernatant fluorescence strongly suggested that the aerosol-treated bacteria experience a loss in membrane integrity, *i.e.*, rupturing, that results in intracellular proteins being released into the surrounding milieu. The net result of this is a dramatic loss in viability, as demonstrated here by the CFU enumeration and flow cytometry analyses, with nearly 50% of the aerosol dispersed *E. coli* dying as a direct result of this treatment.

## Methods

### Bacterial growth

*E. coli* XL1-Blue was obtained from RBC Biosciences, Korea (HIT-Blue Competent Cells, Cat# RH117). This strain was initially grown up on Luria-Bertani (LB) (BD Difco, USA) agar plates (1.6% agar, BD Difco, USA) from which colonies were inoculated into 3 ml LB broth in a 15 ml conical tube (SPL, Korea). These cultures were cultivated at 37 °C with shaking at 250 rpm for 16 hours. Consequently, 25% glycerol stocks were prepared and these were stored at −80 °C. Before preparation of the biofilm, the glycerol stock was streaked on an LB agar plate and incubated overnight at 37 °C to cultivate colonies. From the plate, a single colony was inoculated into 10 ml sterile LB broth in a 50 ml conical tube containing 100 μg/ml ampicillin and incubated with shaking (250 rpm) at 37 °C overnight. To generate a fluorescent variant of XL1-Blue, this strain was transformed with the pAMCyan plasmid (Clontech, USA), which confers resistance to ampicillin.

### E. coli biofilm formation on silicon chips

Initially, silicon chips (10 × 10 mm, Shin-Etsu, Japan) were cleaned by piranha solution (hydrogen peroxide (30%):sulfuric acid (96%) =1:1 v/v) for 10 minutes, rinsed with deionized water for 10 minutes, and then dried under pure nitrogen gas. The contact angles of a distilled water droplet on the chips and surface roughness of the chips were 17.0 ± 1.5 degrees and 22.6 ± 0.8 nm, respectively^20^. As such, this silicon surface is very smooth, which generally facilitates the initial attachment of bacteria^28^. Moreover, this aerosol technique was quite insensitive to the substrate materials employed with regard to the biofilm removal efficiencies^20^. Before preparation of the biofilm, the silicon chips were treated with 70% ethanol for 5 seconds, rinsed in autoclaved deionized water for 5 seconds, and then finally rinsed three times in LB broth for 5 seconds each.

To grow the biofilms, an overnight grown culture of *E*. *coli* XL1-blue (OD_600_2.1) was initially diluted 100-fold into fresh LB media, and 180 μl of this diluted culture was further diluted into 5 ml of fresh LB media. This culture contained an average of 240000 (±39600) viable cells per ml and was poured into a 35 mm Petri dish. Two chips were aseptically placed into each dish and incubated for 24 h at 30 °C without shaking.

### Biofilm removal via CO_2_ aerosol treatment

**Figure 5:**
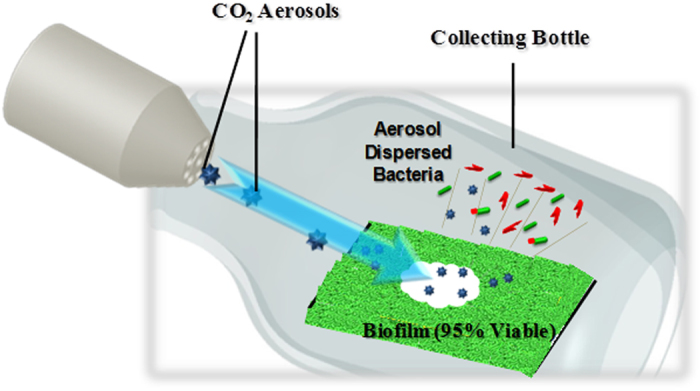
Schematic illustration of the system used in this study to expose bacterial biofilms to CO_2_ aerosols and capture dispersed cells^29^.

The general experimental procedures employed were published previously^19^,^20^. Briefly, the biofilms on the silicon chips were washed gently with 10 mM ammonium acetate buffer (Sigma-Aldrich, USA), and were treated with the aerosols immediately. We placed the *E. coli* biofilm grown chips horizontally and positioned 2 cm from the nozzle in an aerosol flow. The nozzle axis was maintained at 40° angle relative to the chip surface. The bacteria dispersed by the aerosol treatment were collected using a 200 ml bottle arranged over the chip to minimize loss (Fig. 5). A dual gas unit (K6-10DG; Applied Surface Technologies, NJ, USA) was used for aerosol generation. The N_2_ gas pressure was 0.7 MPa and the CO_2_ gas pressure was 5.6 ± 0.2 MPa. The CO_2_ aerosols were off for 3 seconds during each 8-second cleaning cycle, and the total cleaning time was 5 cycles. The average room temperature was 25.2 (±2.1) °C, and the average relative humidity was 67.5 (±7.3) % during all aerosol treatments.

### Enzymatic disruption of the biofilms

The bacteria collected in 25 mM HEPES buffer (pH 7.2) (Sigma-Aldrich, USA) were subjected to enzymatic treatment with Proteinase K (100 ng/ml, Invitrogen, USA) and DNase I (100 ng/ml, Sigma-Aldrich, USA) for 2 h at 37 °C to disrupt any cell clumps that may be present. Likewise, biofilms still present on the treated chips and those on control chips, *i.e.*, untreated, were also dispersed using the same enzymatic treatment.

### Confocal and scanning electron microscopic analysis

Confocal microscopy and SEM were used to visualize the *E. coli* XL1-Blue biofilms that formed on the silicon chips. The bacterial cells were stained by immersing the chip for 30 min in 25 mM HEPES buffer containing BacLight stain (SYTO9-PI, Invitrogen, USA). The chips were then washed gently to remove any excess dye before imaging using an LSM700 confocal microscope (Carl Zeiss) operated by ZEN 2009 software.

Before SEM imaging, the bacteria were fixed by the chemical fixation procedure described by Dwidar *et al.*^29^. The samples were then placed in a critical point dryer (SPI Supplies) for the drying at 35 °C at 1200 psi. Finally, the samples were coated with platinum and imaged with a scanning electron microscope (S-4800, Hitachi).

### Enumeration of viable bacteria

For viability measurements, we determined the CFU of three samples separately: 1) from untreated chips using an enzymatic treatment (Control); 2) the bacteria dispersed by the aerosols (Aerosol); and 3) the bacteria dispersed by the aerosols and then an enzymatic treatment of the same chip to obtain any biofilm-associated bacteria still attached to the chip (Aero + Chip). After aerosol treatment, the bacterial samples were collected with 10 ml of buffer as mentioned above and transferred to a 15 ml conical bottom tube (BD Falcon, USA). For the Aero + Chip samples, the treated chip was added to the collected sample in the same tube to disperse the biofilm attached to the surface using Proteinase K and DNase I as described above. The same protocol was used for the Control, so each of the different samples was present within 10 ml of HEPES buffer (25 mM). The samples were centrifuged at 16,000 g for 5 minutes and the supernatant was used for fluorescence determination. The resulting bacterial pellet was re-suspended in 9.8 ml HEPES buffer, and the number of viable bacteria within each sample was determined by spreading serial dilutions out on LB agar plates and growth of the colonies overnight at 37 °C.

### Fluorescence measurements

From the 10 ml samples obtained above, a small aliquot was taken for fluorescence measurement. The total fluorescence was determined using 200 μl of the collected samples. In parallel, a 200 μl cell-free supernatant sample was also tested to determine the extracellular fluorescence. These samples were prepared by centrifuging the samples as described above and filtering them through sterile 0.22 micron syringe filters to remove any remaining bacterial cells. Likewise, the resulting cell pellet was re-suspended in the same volume of HEPES buffer (25 mM) and the fluorescence of this sample (pellet) was also determined, representing the cell-associated fluorescence. The fluorescence measurement in each case was performed using a 200 μl sample with 96 well black plates (SPL, South Korea) and a fluorescence plate reader (Infinite® 200 PRO – Tecan, Germany). The excitation and emission wavelengths were set to 410 nm and 495 nm, respectively. The fluorescence from each of the samples was normalized using a standard curve and is shown relative to the whole sample fluorescence obtained from the Control.

### Flow cytometry analysis of dispersed bacteria

Each sample was run for 10 seconds using a FACS calibur flow cytometer (BD Biosciences, San Jose, CA), and the number of *E. coli* cells, both viable and dead, passing through the FSC/SSC gated region were counted. For live/dead discrimination, dead bacteria were prepared by adding 50 μl of disinfectant Extran MA 02 (Merck KGaA, Germany) to 450 μl of an overnight bacterial culture and incubating for 30 min at room temperature. Live cells were directly taken from overnight grown suspension. Both samples were stained with BacLight stain (SYTO9 and PI) prior to FACS analysis. The stain exhibits green fluorescence in all bacterial cells, while PI only stains dead bacteria with a red fluorescence. The different population were gated and gate settings were used for analysis of dispersed bacteria from biofilm.

Similarly, the dispersed biofilm samples were stained and underwent FACS analysis as mentioned above. All the samples were kept at room temperature for 10 minutes before the flow cytometry analysis. Experiments were performed in a BD LSRFortessa Flow Cytometer (BD Biosciences, San Jose, CA) using a blue (488 nm) laser to excite the stained cells. The green fluorescence emission was detected with a 530/30 band pass filter and the red fluorescence emission was detected with a 695/40 band pass filter. The fluorescence emission was acquired for 5,000 cells and displayed in an exponential scale using BD FACS Diva v.6.2 (BD Biosciences San Jose, CA).

### Data analysis

Each of the experiments was performed independently in triplicate for error analysis. The average values obtained are shown in the figures with the standard deviations presented as the error bars. Statistical analyses comparing the results amongst the different treatments were performed using a one-way ANOVA test followed by the Tukey post hoc test. Significantly different results are designated with asterisks (*) within the corresponding figures.

## Additional Information

**How to cite this article**: Singh, R. *et al.* Effects of Carbon Dioxide Aerosols on the Viability of *Escherichia coli* during Biofilm Dispersal. *Sci. Rep.* **5**, 13766; doi: 10.1038/srep13766 (2015).

## Supplementary Material

Supplementary Information
