# Supplementary material for: Effects of Carbon Dioxide Aerosols on the Viability of Escherichia coli during Biofilm Dispersal
Source: Sci Rep. 2015 Sep 8;5:13766. doi: 10.1038/srep13766 (PMC4561891; doi:10.1038/srep13766)
Supplement: Supplementary Information [file srep13766-s1.pdf]

Supplementary Information

Effects of Carbon Dioxide Aerosols on the Viability of *Escherichia coli* during Biofilm Dispersal

Renu Singh[1†], Ajay K. Monnappa[2†], Seongkyeol Hong[1], Robert J. Mitchell[2]*, Jaesung Jang[1]*

† - These authors contributed equally.

[1]School of Mechanical and Nuclear Engineering, Ulsan National Institute of Science and Technology (UNIST), Ulsan 689-798, S. Korea

[2]School of Life Sciences, Department of Biological Sciences, Ulsan National Institute of Science and Technology (UNIST), Ulsan 689-798, S. Korea

***Corresponding Author Information**

Robert J. Mitchell: email – esgott@unist.ac.kr; Tel - +82-52-217-2513; Fax - +82-52-217-2509

Jaesung Jang: email – jjang@unist.ac.kr; Tel - +82-52-217-2323; Fax - +82-52-217-2409

S1)

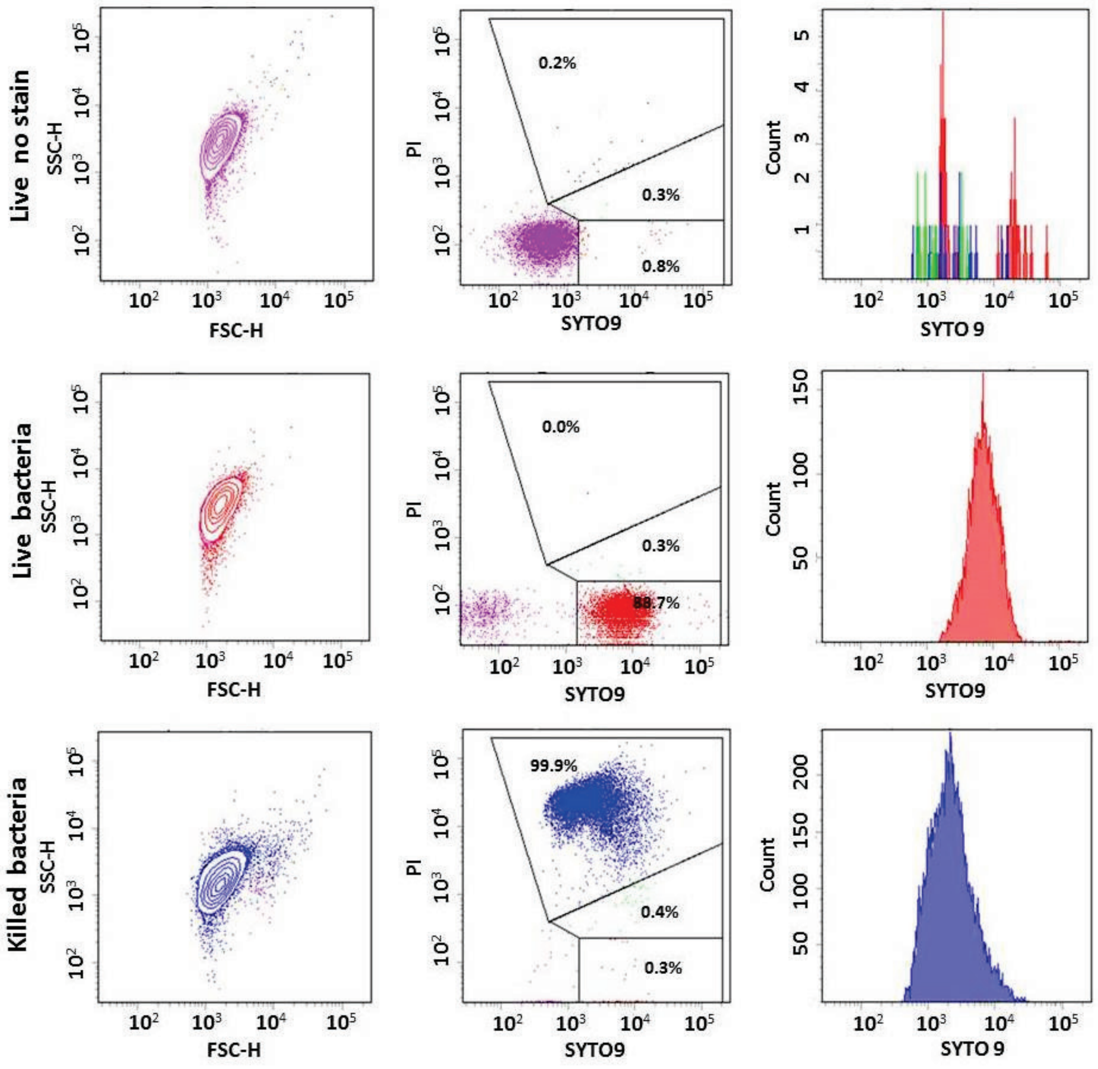

Fig. S1 Live dead discrimination of *E. coli* using FACS. For Live dead discrimination, live cells were directly taken from overnight bacterial culture. Dead bacteria were prepared by adding 50µl of Extran MA 02(Merck KGaA, Germany) to 450µl of overnight grown bacterial culture and incubating for 30 min at room temperature. Both killed and live bacteria were stained with BacLight stain (SYTO9 and propidium iodide) prior to FACS analysis. Unstained bacteria were used for gating Stained population. A total of 5,000 cells were analyzed for gating live, dead, and injured population.

S2)

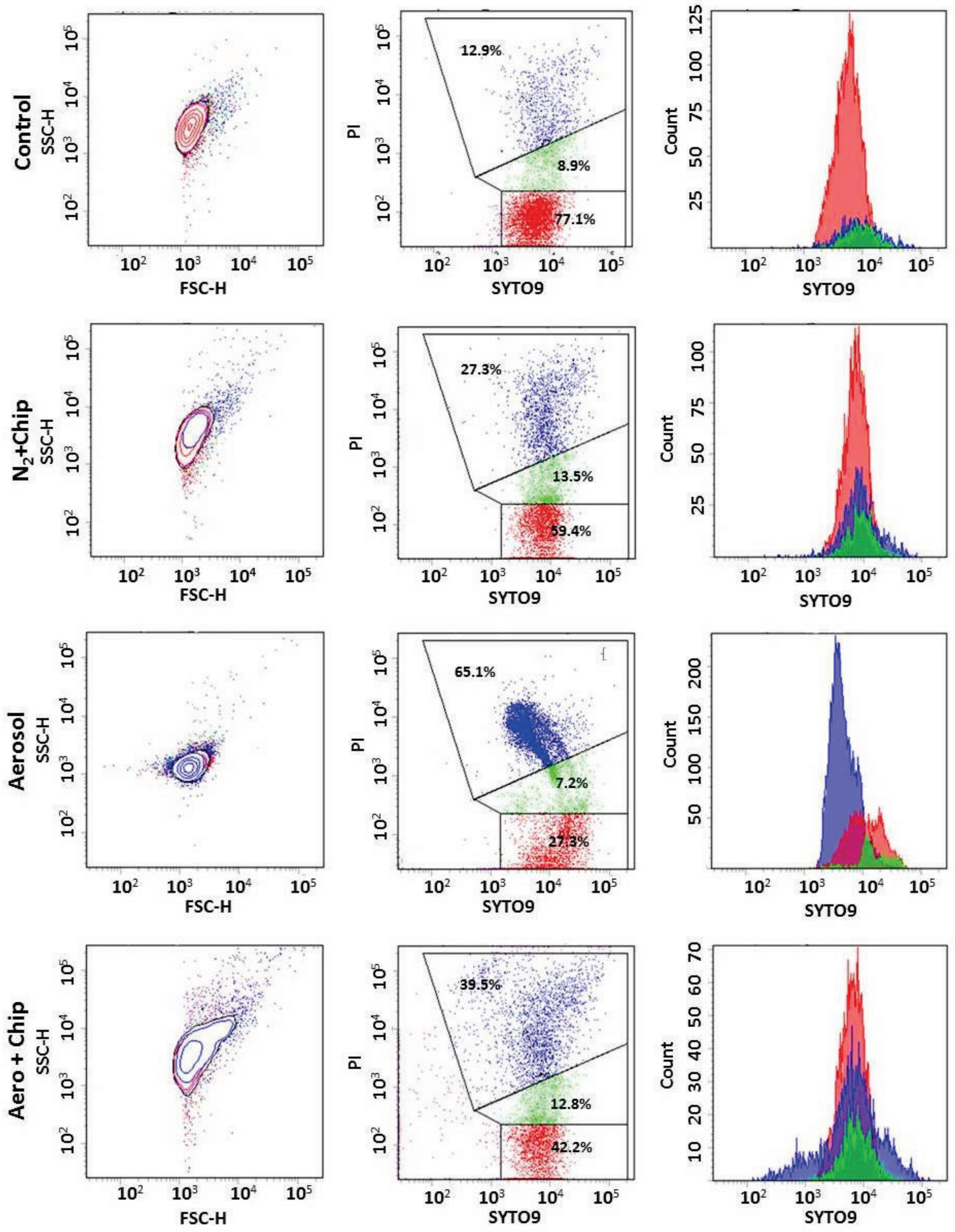

Fig. S2 Representative dot plot from a single FACS analysis. For each of the 4 samples, i.e., Control, N_2+ Chip, Aerosol, and Aero + Chip, 5,000 events were analyzed to determine live, dead and injured population.
